# Intrinsic deuterium labeling of spinach: a hydroponic method to enrich isotopic labeling of vitamins E and K suitable for human bioavailability studies

**DOI:** 10.3389/fpls.2026.1915355

**Published:** 2026-10-14

**Authors:** Shahabeddin Rezaei, Sisi Cao, Min Zeng, Maria L. Antonius, Allyson Q. McHenry, Richard S. Bruno, Joshua J. Blakeslee

**Affiliations:** 1 Human Nutrition Program, Department of Human Sciences, The Ohio State University, Columbus, OH, United States; 2 Department of Horticulture and Crop Science, The Ohio State University, Columbus, OH, United States; 3 Laboratory for the Analysis of Metabolites from Plants (LAMP), The Ohio State University, Columbus, OH, United States

**Keywords:** hydroponic system, intrinsic labeling, spinach, vitamin E, vitamin K

## Abstract

**Introduction:**

Intrinsic stable isotope labeling enables assessment of nutrient bioavailability from intact plant food matrices by distinguishing nutrients derived from the test food from existing body pools. Although deuterium oxide (D_2_O) has been used to intrinsically label green leafy vegetables under hydroponic growth conditions, well-characterized protocols for static hydroponic systems remain limited.

**Methods:**

This study established and evaluated a novel hydroponic D_2_O-labeling protocol using spinach to produce intrinsically labeled α-tocopherol (α-T) and phylloquinone (VK1). Three independent experiments were conducted in a controlled-environment growth chamber. Spinach seedlings were transferred to a static hydroponic system containing 0% (control) or nominal 30% (v/v) D_2_O nutrient solution. Plants grown under controlled environmental conditions were harvested at Weeks 4, 5, and 6 to evaluate leaf biomass, nutrient content, and isotopomer distributions.

**Results:**

No labeling condition × harvest time interactions occurred for leaf biomass, α-T content, or VK1 content (*p* > 0.05). Leaf biomass, α-T content, and VK1 content increased from Week 4 to Week 6 (*p* < 0.05) but were unaffected by labeling condition (*p* > 0.05). Compared with non-D_2_O-grown spinach, D_2_O-grown spinach demonstrated significantly higher relative abundance of the summed d_3_–d_20_ mass isotopomers for α-T (98.7–99.0% vs 3.6–4.6%; *p* = 0.0002) and VK1 (97.0–97.5% vs 1.8–2.1%; *p* = 0.0008). The dominant labeled isotopomers were d_9_ for α-T and d_8_–d_9_ for VK1. Inter-experiment coefficients of variation for the summed d_3_–d_20_ relative abundance ranged from 0.1–0.3% for α-T and 0.1–0.4% for VK1 across harvest times.

**Discussion:**

These findings provide a detailed framework for a static hydroponic D_2_O-labeling method that produced similar α-T and VK1 isotopomer distributions across three experiments conducted in the same laboratory, with potential application in future bioavailability and metabolic tracing research.

## Introduction

Green leafy vegetables, such as spinach, are rich in fat-soluble nutrients including vitamin E (α-tocopherol; α-T) and vitamin K_1_ (phylloquinone; VK1) (Shearer et al., 2012; Chun et al., 2006). However, nutrient content alone does not indicate how much these foods contribute to vitamin E and K nutriture in humans. Indeed, the bioavailability or the fraction of an ingested nutrient that is absorbed and becomes available for physiological use is influenced by food- and host-related factors, including the plant matrix and individual pre-existing nutrient stores (Parada and Aguilera, 2007). Thus, defining the nutritional value of green leafy vegetables requires approaches that directly quantify nutrient bioavailability from the intact plant matrix with discrimination of food-derived nutrients from endogenous pools.

Intrinsic stable isotope labeling of plants provides a rigorous method to assess nutrient bioavailability from whole foods (Grusak, 1997). In this approach, low-abundance stable isotopes (e.g., deuterium, carbon-13) are incorporated into plant metabolites during biosynthesis, ensuring that labeled nutrients remain embedded within the native plant matrix and follow physiological digestive and metabolic pathways. This strategy overcomes limitations inherent to dietary supplement-based studies, which bypass food matrix effects, and non-labeled feeding studies that cannot differentiate test-food–derived nutrients from background pools (Grusak, 1997).

For vitamins E and K, intrinsic labeling has been achieved using atmospheric carbon-13 dioxide (^13^CO_2_) or deuterium oxide (D_2_O) under hydroponic growth conditions (Dolnikowski et al., 2002; Ellis et al., 2019; Novotny et al., 2010; Erkkilä et al., 2004; Fu et al., 2009; Kurilich et al., 2003; Traber et al., 2015). Atmospheric ^13^CO_2_ labeling yields highly enriched compounds with a single dominant isotopomer, facilitating bioanalytical quantification, but requires specialized sealed growth chambers and gas-delivery systems with carefully regulated flow-rates (Novotny et al., 2003; Ćeranić et al., 2020; Peters et al., 2018; Gleichenhagen et al., 2013), which increases complexity and cost (Novotny et al., 2003; Grusak, 1997). In contrast, D_2_O labeling is more accessible and cost-effective, as it does not require atmospheric control (Grusak, 1997). However, because plant growth is impaired at high D_2_O enrichments, labeling is typically limited to ~30 atom% D_2_O, producing a broad isotopomer distribution rather than a single labeled mass (Katz and Crespi, 1966). Although this increases analytical complexity, it can be addressed through pre-characterization of isotopomer profiles and appropriate experimental design (Grusak, 1997).

Using D_2_O-based intrinsic labeling, green leafy vegetables such as spinach, collards, and kale have been intrinsically labeled to investigate the bioavailability of α-T (Traber et al., 2015), VK1 (Ellis et al., 2019; Fu et al., 2009; Erkkilä et al., 2004), lutein (Lienau et al., 2003), and β-carotene (Tang et al., 2005; Muzhingi et al., 2017) in humans. As summarized in **Table 1**, these studies typically employ hydroponic growth systems housed in controlled environments. They consist of static systems having independent plant growth containers with direct aeration, or recirculating systems, in which nutrient solution is continuously circulated through interconnected reservoirs (Grusak and Pezeshgi, 1994; Grusak, 1997; Weaver and Chaney, 1985).

**Table 1:** Intrinsically deuterium-labeled leafy vegetable models used in clinical studies of fat-soluble compounds bioavailability.

Reference	Plant species/cultivar	Target compound	D_2_O concentration	Germination method	Hydroponic system (static vs recirculating)	Harvest time	Dominant isotopomer	Cited method reference(s)
Current study	Spinacia oleracea L. cv. Platypus RZ F1	α-T	30% (v/v), nominal	Rockwool	Static	Week 4-6	Week 4: D_9_ Week 5: D_9_ Week 6: D_9_	–
Current study	Spinacia oleracea L. cv. Platypus RZ F1	VK1	30% (v/v), nominal	Rockwool	Static	Week 4-6	Week 4: D_9_ Week 5: D_8_ Week 6: D_8_	–
(Tang et al., 2005)	Spinacia oleracea L. cv. Melody	β-carotene	25 atom%	NR	NR	NR	D_10_	(Grusak, 1997)
(Lienau et al., 2003)	Spinacia oleracea L. cv. Melody	Lutein	25 atom%	Filter Paper	NR	Week-4	D_8_^a^	(Grusak, 1997)
(Ellis et al., 2019)	Brassica oleracea L. var. acephala cv. Georgia	VK1	31 atom%	NR	NR	NR	D_10_	(Erkkilä et al., 2004)
(Traber et al., 2015)	Brassica oleracea L. var. acephala cv. Georgia	α-T	31 atom%	NR	NR	Week-6	D_11_^b^	(Erkkilä et al., 2004)
(Fu et al., 2009)	Brassica oleracea L. var. acephala (cultivar NR)	VK1	NR	NR	NR	NR	D_10_^c^	(Dolnikowski et al., 2002)
(Erkkilä et al., 2004)	Brassica oleracea L. var. acephala cv. Georgia	VK1	31 atom%	NR	NR	Week 6	D_9_^d^	(Dolnikowski et al., 2002); (Grusak, 1997); (Grusak and Pezeshgi, 1994)
(Lienau et al., 2003)	Brassica oleracea L. var. acephala cvs. Grady Simpson and Bluemax	Lutein	25 atom%	Filter paper	NR	Week 6	D_8_^e^	(Grusak, 1997)
(Muzhingi et al., 2017)	Brassica oleracea L. var. acephala (cultivar NR)	*β*-carotene	NR	NR	NR	NR	D_9_	(Tang et al., 2005); Erkkilä et al., 2004)

NR, Not Reported; α-T, α-Tocopherol; VK1, Phylloquinone.

^a^ Using liquid-chromatography mass spectrometry (LC–MS) with atmospheric-pressure chemical ionization (APCI) operated in positive-ion mode, the most abundant isotopomers were m/z 556–562 (D_5_–D_11_), which accounted for 63% of the total lutein isotopomer abundance, calculated as the summed ion abundances for m/z 556–562 (D_5_–D_11_) relative to the summed total ion abundances across m/z 552–569 (D_1_–D_18_).

^b^ Using LC-MS with APCI operated in negative-ion mode, the most abundant labeled isotopomers ranged from m/z 438–442 (D_9_–D_13_) and accounted for approximately 60% of the total α-tocopherol isotopomer abundance, calculated as the summed ion abundances for m/z 438–442 relative to the summed total ion abundances across all detected α-tocopherol isotopomers (full m/z range not reported).

^c^ Using LC–MS with APCI operated in positive-ion mode, the most abundant labeled isotopomers ranged from m/z 459–463 (D_8_–D_12_) and accounted for 64% of the total VK1 isotopomer abundance, calculated as the summed ion abundances for m/z 459–463 relative to the summed total ion abundances across all detected VK1 isotopomers. Although the full m/z range was reported as 450–470, the lower bound is inconsistent with positive-ion mode, as the unlabeled isotopomer of VK1 would be expected at m/z 451; therefore, the full range could not be determined for this study.

^d^ Using GC–MS with methane negative chemical ionization, the unlabeled VK1 isotopomer was detected at m/z 450, and the dominant labeled isotopomers ranged from m/z 459–462 (D_9_–D_12_), accounting for 37% of the total labeled VK1 isotopomer abundance, based on the reported ratio of summed ion abundances for m/z 459–462 relative to m/z 453–469.

^e^ Using LC–MS with APCI operated in positive-ion mode, the most abundant isotopomers were m/z 556–562 (D_5_–D_11_), which accounted for 48% of the total lutein isotopomer abundance, calculated as the summed ion abundances for m/z 556–562 (D_5_–D_11_) relative to the summed total ion abundances across m/z 552–569 (D_1_–D_18_).

Static systems are often described as suitable for applications involving smaller quantities of plants or temporary set-ups (Grusak, 1997; Weaver and Chaney, 1985), whereas recirculating systems are better suited for longer-term growth and labeling (Weaver and Chaney, 1985; Grusak and Pezeshgi, 1994). However, not all investigators have the infrastructure to implement recirculating hydroponic systems, which can also increase the costs of labeling due to the larger volumes of liquid media required for recirculation. Published intrinsic D_2_O-labeling studies in green leafy vegetables (**Table 1**) provide limited methodological detail or refer to previously published cultivation methods, including protocols developed for other plant species or recirculating systems (Dolnikowski et al., 2002; Grusak and Pezeshgi, 1994). Although selected environmental conditions, nutrient formulations, and harvest times have been reported, key operational details—including methods for seed germination, hydroponic system configuration, the number of plants within the cultivation system, nutrient-solution volume, aeration setup, replenishment procedures, and procedures to accommodate plant development—have often been presented incompletely or omitted from previously published studies (Lienau et al., 2003; Tang et al., 2005; Erkkilä et al., 2004; Traber et al., 2015; Ellis et al., 2019).

D_2_O-based labeling has also been used in plants for applications other than human bioavailability research, including structural characterization of plant biomass and investigation of metabolic and biosynthetic processes (Osipenko et al., 2023; Evans et al., 2019, 2014, 2015; Tamogami et al., 2013; Åstot et al., 2000; Nett et al., 2018; Tat and Lee, 2024). These protocols were developed for different plant species, analytical objectives, and experimental scales. Biomass-focused studies used specialized perfusion or small-scale hydroponic systems, whereas metabolic studies generally used liquid culture, tissue-feeding, vials, or Petri dishes. Consequently, their cultivation configurations and resource requirements differ from those of the present study.

Taken together, studies supporting human bioavailability research lacked sufficient operational detail, whereas the non-clinical protocols were developed for different analytical objectives and cultivation requirements. We did not identify a sufficiently detailed static hydroponic D_2_O-labeling protocol that could be directly implemented or adapted. The present study addresses this gap by describing a static system that uses a glass-bead matrix to support root development while accommodating approximately 100 spinach plants with an initial nutrient-solution volume of 0.7 L. Humidity domes and a dedicated replenishment port were incorporated to limit D_2_O loss during cultivation. The protocol was also evaluated across three independent experiments to assess the inter-run precision of α-T and VK1 isotopomer enrichment.

The objective of this methods report was to develop and describe a static hydroponic D_2_O labeling protocol using spinach as a plant model. This protocol builds on previous studies but uses a solid support system to reduce evaporative loss of deuterated media. Additionally, the protocol outlines methodological details often unclear in previous works, including methods for seed germination, plant position with the hydroponic system, aeration setup, and replenishment procedures. The inter-run precision of the protocol was assessed by quantifying α-T and VK1 isotopomer enrichment across three independent experiments. Spinach leaf biomass, α-T and VK1 content, and isotopomer distributions were measured to inform dose selection and confirm deuterium incorporation beyond natural isotope abundance. Given its high content of fat-soluble nutrients and short growth cycle, as well as its minimal requirements for hydroponic inputs, spinach provides an effective model for developing intrinsically labeled plant foods. This work provides a framework for future bioavailability and metabolic-tracing studies of plant-derived nutrients from intact plant foods.

## Materials and methods

### Plant materials

Platypus RZ F1 spinach seeds were obtained from Rijk Zwaan (De Lier, Netherlands). D_2_O (99.8 atom% purity) was purchased from Sigma-Aldrich ISOTEC (Miamisburg, OH, USA). Rockwool sheets (2.5 × 2.5 × 3.8 cm; AO25/40 Cubes), seed trays without drainage holes (26.7 × 53.3 cm), humidity domes (5.08 cm and 17.78 cm), Hydro-Gro Vine fertilizer, calcium nitrate fertilizer, and zinc (Zn) sulfate fertilizer were purchased from CropKing, Inc. (Lido, OH, USA). 2-(N-morpholino)ethanesulfonic acid (MES, anhydrous) was purchased from Research Products International (Mount Prospect, IL, USA).

### Preparation of non-D_2_O and D_2_O hydroponic nutrient solutions

Non-D_2_O hydroponic nutrient solution contained 100% deionized water, whereas D_2_O-enriched hydroponic nutrient solution contained nominal 30% (v/v) D_2_O with deionized water. A 30% (v/v) D_2_O concentration was selected based on prior studies demonstrating effective intrinsic labeling without significant impairment of plant growth or development (Katz and Crespi, 1966; Grusak, 1997).

For each 2.0 L of prepared solution, 5.94 g Hydro-Gro Vine fertilizer, 3.96 g calcium nitrate fertilizer, and 0.39 g MES (buffering agent) were added sequentially and manually mixed until all solutes were fully dissolved. Hydro-Gro Vine fertilizer contained 4.4% total nitrogen, 13% available phosphoric acid, 34% soluble potash, and 3.7% magnesium, along with trace elements including iron (1000 mg/kg), manganese (800 mg/kg), Zn (270 mg/kg), copper (95 mg/kg), boron (275 mg/kg), and molybdenum (40 mg/kg). Calcium nitrate fertilizer contained 1.0% ammonia calcium nitrate, 14.5% nitrate nitrogen, and 19.0% water-soluble calcium. Hydroponic growth solution pH was adjusted to 5.3 using 8 M potassium hydroxide or 3 N hydrochloric acid and monitored for 10 min using a calibrated pH meter (Bluelab Guardian Monitor; CropKing, Inc., Lodi, OH, USA) to confirm buffering capacity. Finally, 20 µL of a supplemental zinc solution (zinc sulfate monohydrate; 36% Zn), prepared at a concentration of 1.44 mg/mL, was added to the solution.

### Seed germination

Spinach seeds were surface-sterilized using chlorine gas to minimize microbial contamination (Lindsey Iii et al., 2017). Seeds were placed in an open sterile polystyrene Petri dish (60 × 15 mm; VWR, PA, USA) and transferred to a glass desiccation chamber (**Figure 1**). Chlorine gas was generated within the sealed desiccator by combining approximately 50 mL of Clorox^®^ Germicidal Bleach (8.25% sodium hypochlorite) with approximately 1 mL of concentrated hydrochloric acid (36.5–38.0% HCl; MilliporeSigma, MA, USA) in a 100 mL glass beaker. Seeds were exposed to chlorine gas for 45 min inside a sealed desiccator placed within a fume hood.

**Figure 1:**
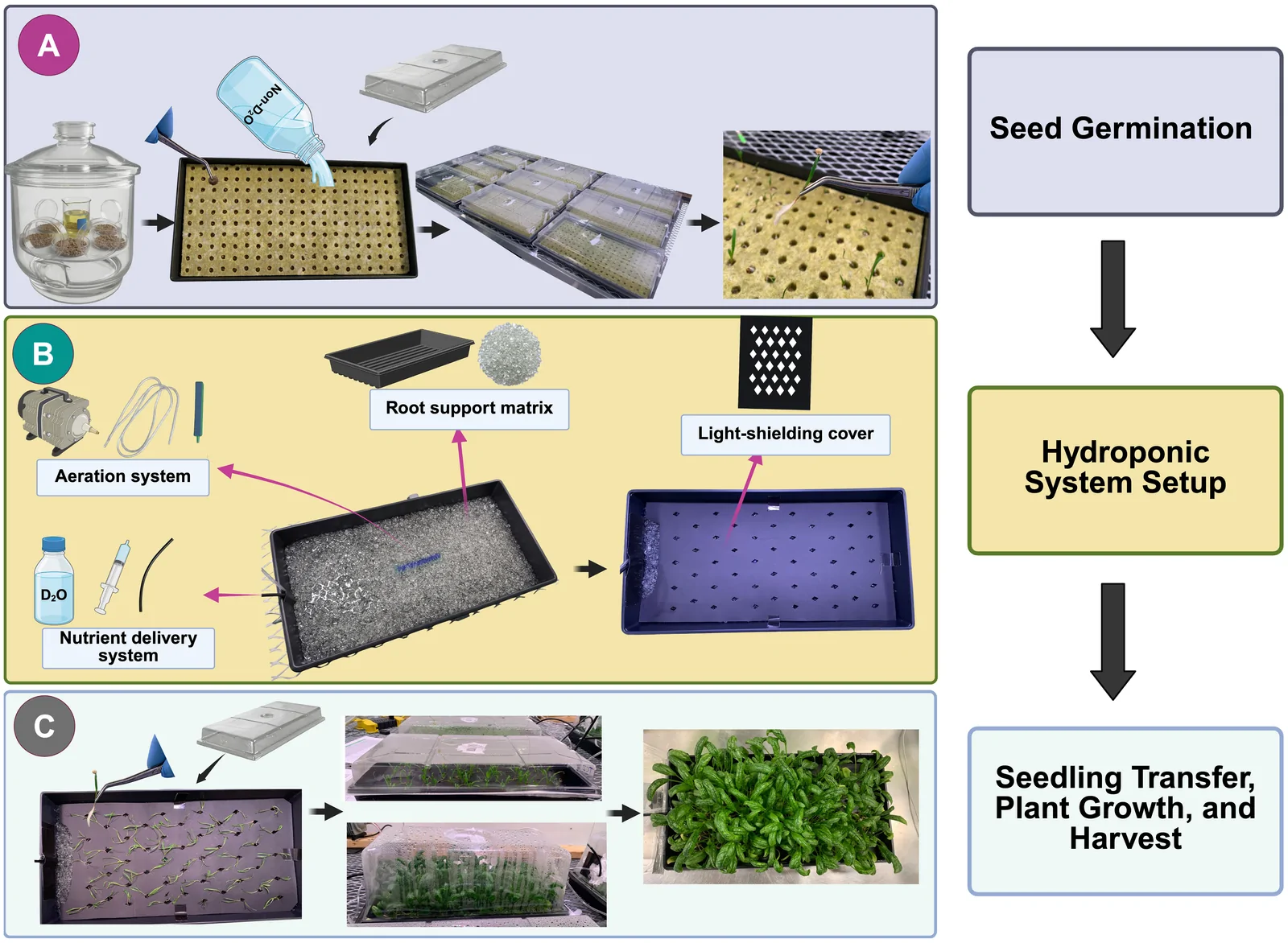
Schematic overview of the static intrinsic D_2_O labeling method used to label α-tocopherol and phylloquinone in spinach. **(A)** Spinach seeds were surface-sterilized using chlorine gas, implanted into rockwool sheets, and irrigated with non-D_2_O hydroponic nutrient solution to initiate germination. **(B)** A non-recirculating, non-interconnected hydroponic system was used, in which plants were grown in individual trays filled with glass beads to support root development. Each tray was continuously aerated using an air pump to provide oxygen to the roots and included a light-blocking black plastic sheet to minimize algal growth in the hydroponic media. Approximately 700 mL of hydroponic nutrient solution was added to each tray at initiation. Hydroponic solution volume was monitored throughout growth and replenished as needed via black silicone tubing. **(C)** At day 7, germinated seedlings were transferred to hydroponic nutrient solution containing nominal 30% (v/v) D_2_O for intrinsic labeling. A 5.08 cm high humidity dome was used during early growth and was replaced with a 17.78 cm high humidity dome to accommodate plant growth. Created in BioRender. Rezaei et al. (2026) https://BioRender.com/3shn20f.

Following sterilization, the desiccator was vented, and the Petri dish was immediately shut, wrapped with 3M™ Micropore™ tape (2.54 cm width; Fisher Scientific, Florence, KY, USA), and transferred to the walk-in growth chamber housing the hydroponic growth set-up. A total of 200 surface-sterilized seeds were implanted into an individual rockwool sheet (2 seeds per hole) (see below, “Hydroponic System Setup”), which was then saturated with 2 L of non-D_2_O hydroponic nutrient solution. Trays were covered with humidity domes (5.08 cm height) and maintained under controlled growth conditions as described below. After 7 days, germinated seedlings were removed from the rockwool sheet and transferred to trays containing D_2_O-enriched nutrient solution for intrinsic labeling.

### Hydroponic system setup for intrinsic D_2_O labeling

To generate spinach leaf tissue, a hydroponic system was used, in which plants were grown in individual, non-interconnected trays (**Figure 1****).** Trays were filled to approximately one-third of their depth with clear glass beads (Glacier Ice Size 2 Jelly Bean Glass; American Specialty Glass, UT, USA) to support root development. To reduce microbial contamination, prior to use, beads were washed with 10% (v/v) bleach, detergent, and deionized water; then rinsed thoroughly and soaked in 100% ethanol (200 proof; Decon™ Labs, King of Prussia, PA, USA) for at least 8 h. After cleaning, beads were allowed to air-dry in a sterile laminar flow hood, and then placed in sterilized plastic bins, which were sealed with 3M™ Micropore™ tape until use.

Continuous aeration was provided using an air stone (11.5 × 2 × 1.2 cm; Waycreat, Shenzhen, China) connected to a commercial-grade air pump system (EcoPlus ECOair 5, 1300 GPH, 80 W, single outlet with an 8-valve manifold; Vancouver, WA, USA) via flexible transparent plastic tubing (outer diameter = 6 mm, inner diameter = 4 mm; Penn-Plax Inc., Hauppauge, NY, USA) (**Figure 1**). Aeration was maintained throughout the labeling period to prevent oxygen depletion in the static nutrient solution and to support root respiration (Grusak, 1997).

Approximately 0.7 L of D_2_O hydroponic nutrient solution was added to each tray (actual volume: 732 ± 16 mL, mean ± SEM; n = 6 trays), sufficient to cover the upper layer of glass beads and provide a reference volume for monitoring solution loss. Nutrient solution volume was monitored three times per week and replenished as needed (*i.e.*, volumes of deuterated hydroponic solution were added to bring the total media volume to a level sufficient to cover the upper layer of glass beads). During plant growth, replenishment was performed via black silicone tubing (ALEGI; 4.8 mm internal diameter) inserted through a designated access port in the tray lid and secured to the tray lid with duct tape. The solution was delivered using a 10 mL syringe to minimize tray opening and D_2_O loss (**Figure 1**). Black tubing was used to limit algal growth and the tubing was sealed with Parafilm when not in use.

To further limit light penetration into the hydroponic growth media and subsequent algal growth, the glass bead/hydroponic media system in each tray was covered with a black plastic sheet (55.9 × 71.1 cm poster board; UCreate^®^; Dixon Ticonderoga Company, Appleton, WI, USA), cut to fit tray dimensions and secured with duct tape (**Figure 1**) attached above the media fill line. Fifty holes (~1 × 2 cm) were cut into each sheet in staggered rows to allow seedling transfer. Seedlings were inserted using forceps such that roots were positioned below the plastic sheet with roots being gently inserted into the glass beads (into a small cavity made using the forceps) and aerial tissues (stems, petioles, cotyledons, and any emerging leaves) above the plastic sheet. Two seedlings were placed per hole, for a total of 100 plants per tray.

Following transfer, trays were covered with 5.08 cm high humidity domes to increase relative humidity and reduce D_2_O loss via transpiration (**Figure 1**). During Week 3, domes were replaced with 17.78 cm high humidity domes to accommodate leaf expansion. Over the 6-week growth period, nutrient solution was replenished as needed, with mean additions of 84 ± 5 mL per tray per week for the non-D_2_O condition and 76 ± 11 mL per tray per week for the D_2_O condition (mean ± SEM; n = 3 trays per condition).

### Growing chamber conditions

Spinach was cultivated in a controlled-environment growth chamber under a 16-h light/8-h dark photoperiod (07:00–23:00). Room air temperature was periodically monitored during cultivation and was 21 °C at all monitored time points. These conditions fall within reported optimal ranges for spinach cultivation (Meng et al., 2025; Isaza et al., 2025). Lighting was provided by an LED system with red, white, and blue channels operated at controller settings of R25, W50, and B50, respectively. Bench-level photosynthetic photon flux density ranged from 200–220 µmol m^-2^ s^-1^ across tray positions. Because the chamber lacked integrated humidity control, two portable dehumidifiers (Midea MAD50C1ZWS and Kesnos PD160A) were used to reduce seasonal humidity fluctuations. Chamber relative humidity was periodically monitored and averaged 43.6 ± 0.6% (mean ± SEM; range: 36.5–48.5%) during cultivation. Temperature and relative humidity beneath the humidity domes were not measured and may have differed from chamber conditions.

### Experimental design

An overview of the experimental design is shown in **Figure 2**. Three independent experiments were conducted in separate batches (Batches 1, 2, and 3), with plants grown for six weeks in each batch, to assess the inter-run precision of α-T and VK1 isotopomer enrichment. All experiments were performed in the same growth chamber, under identical conditions, in facilities provided by the Center for Applied Plant Sciences (CAPS), Rightmire Hall, The Ohio State University. Each experiment included one D_2_O tray and one non-D_2_O control tray, for a total of six trays. Each tray represented an experimental unit, resulting in three experimental units per condition. Within each experiment, the two trays were positioned near one another and remained in fixed locations throughout the experiment without randomization or rotation. In each experiment, 100 plants were grown in D_2_O-enriched nutrient solution (nominal 30% [v/v] D_2_O) and 100 plants in non-D_2_O nutrient solution. Harvesting began at Week 4, consistent with prior intrinsic labeling studies (Lienau et al., 2003), and continued through Weeks 5 and 6 (**Figure 3**). At each harvest, five subsamples were collected from each tray, each comprising leaves pooled from six different plants selected from different tray locations. Pooling plants selected from different tray locations was intended to obtain subsamples representative of growth across each tray rather than to characterize individual plant-to-plant variation. The combined fresh leaf mass of each six-plant subsample was recorded immediately using an analytical balance (Scout model CX1201; OHAUS Corporation, Parsippany, NJ, USA). The subsamples were then placed in Mylar bags, transported on dry ice to the OSU Bionutrition Core Laboratory, and stored at −80 °C until analysis of α-T, VK1, and isotopomer distributions. Before bioanalytical analysis, the five subsamples collected from each tray at each harvest were combined to produce one tray-level sample for α-T and VK1 measurement.

**Figure 2:**
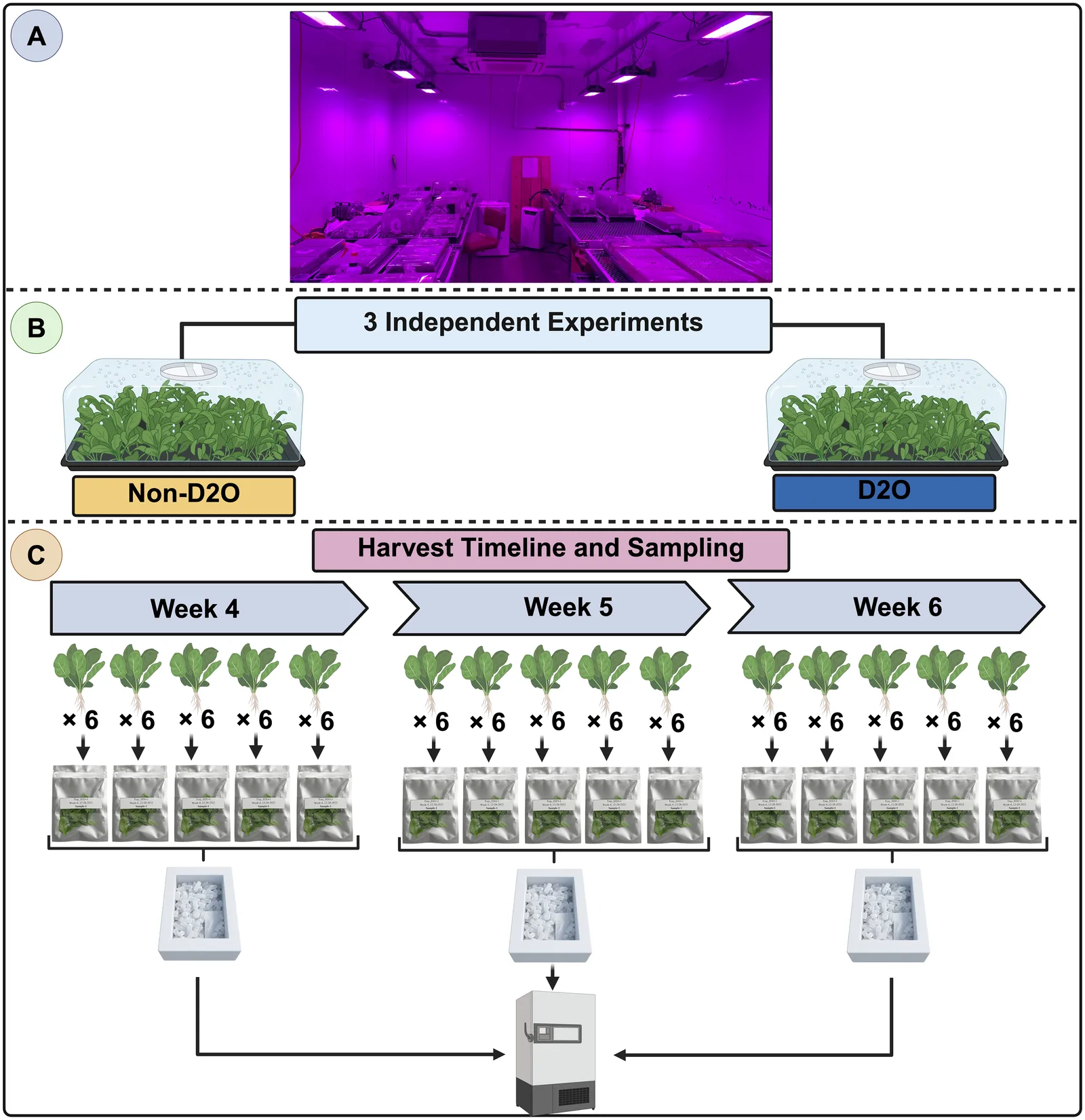
Overview of the experimental design and sampling timeline. **(A)** Spinach was cultivated in an environmentally controlled growth chamber under controlled temperature, humidity, and light conditions. **(B)** Three independent experiments were conducted to assess the inter-run precision of the established intrinsic deuterium oxide (D_2_O) labeling method. Each experiment included one nominal 30% (v/v) D_2_O tray and one non-D_2_O control tray, and each tray represented an experimental unit. **(C)** Harvesting began at Week 4 and continued through Weeks 5 and 6. At each time point, five subsamples were collected per tray, with each subsample comprising pooled leaves from six plants selected from different locations across the tray to better represent overall plant growth and to ensure sufficient biomass for weight measurement and subsequent bioanalytical analyses. Samples were transported on dry ice and stored at −80 °C until analysis. Created in BioRender. Rezaei et al. (2026) https://BioRender.com/3shn20f.

**Figure 3:**
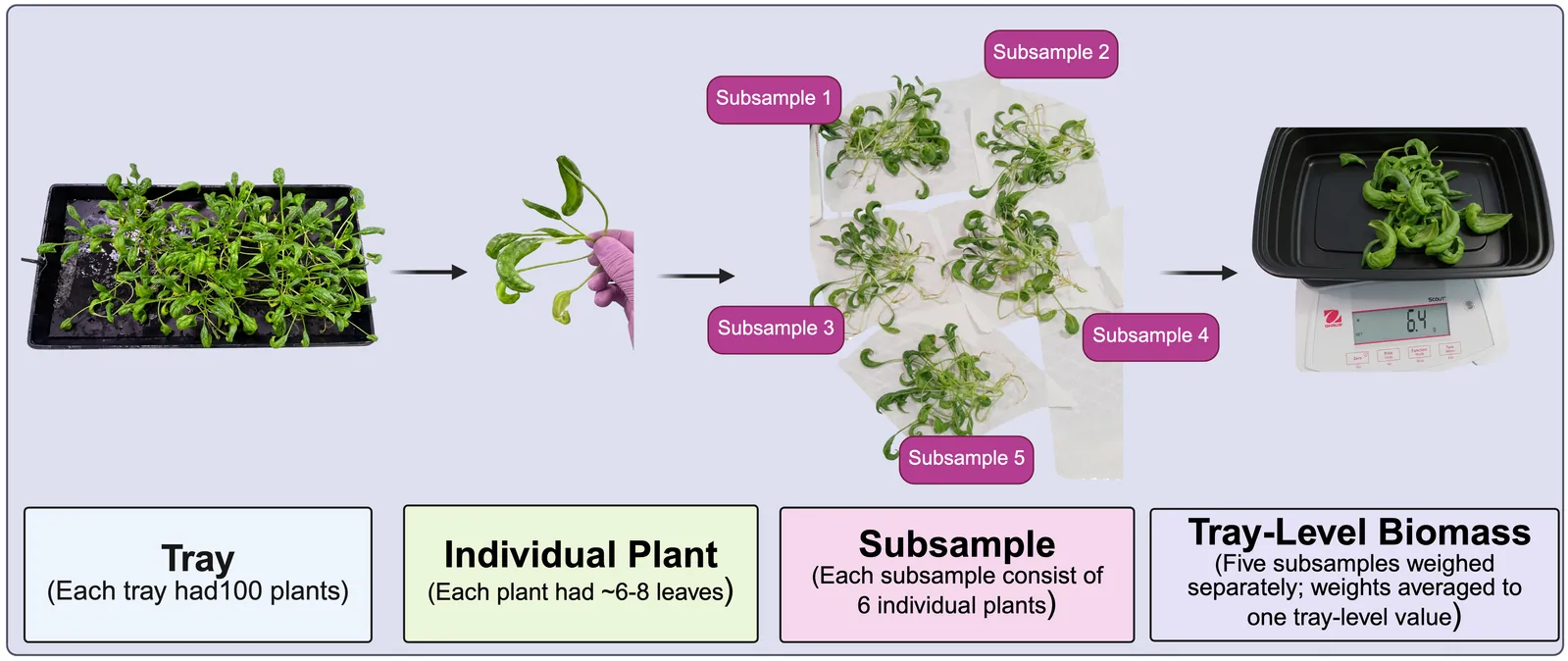
Sampling workflow for spinach leaf biomass measurement. Spinach plants were cultivated in static hydroponic trays containing approximately 100 plants per tray. At each harvest time point (Weeks 4–6), five subsamples were collected per tray. For each subsample, six individual plants were selected from different locations across the tray, and leaf tissue was collected and pooled. The fresh weight of each subsample was recorded immediately after collection using an analytical balance, and the five subsample weights were averaged to obtain one tray-level biomass value at each harvest. Created in BioRender. Rezaei et al. (2026) https://BioRender.com/3shn20f.

### LC-MS analysis of α-T

α-T was extracted and quantified using an established LC-MS method (Mah et al., 2015). Spinach samples were pooled and pulverized with a mortar and pestle under liquid nitrogen. Five analytical replicates were prepared from each pooled sample. Aliquots (100 mg) were saponified at 70 °C with potassium hydroxide in the presence of alcoholic ascorbic acid. Hexane extraction was performed with butylated hydroxytoluene and additional ascorbic acid to prevent oxidation. Extracts were dried under nitrogen, reconstituted in methanol:ethanol (1:1, v/v), and spiked with tocol internal standard (17.8 pmol/uL; #AABH9A955256; Sigma-Aldrich Inc., St. Louis, MO, USA). Samples (2 µL) were analyzed on a Shimadzu LCMS-2020 single-quadrupole system equipped with an APCI source operated in negative-ion mode. Isocratic separation was achieved using 100% methanol (0.3 mL/min) on a Kinetex C18 column (100 × 2.1 mm, 2.6 µm, 100 Å) at 30 °C. The interface, desolvation line, and heat block temperatures were set to 300 °C, 250 °C, and 200 °C, respectively, and the nebulizing and drying gas flows were set to 4 L/min and 6 L/min, respectively. Single ion monitoring was performed at *m/z* 429–449 (d_0_–d_20_). α-T concentrations were calculated from summed isotopomer peak areas relative to external standards and normalized to the internal standard. Measurements from the five analytical replicates were averaged to obtain one tray-level α-T value; these replicates did not contribute to the biological sample size.

### LC-MS analysis of VK1

VK1 was quantified using an established LC-MS method based on Fu et al (Fu et al., 2009), with minor modifications. Aliquots of pulverized samples (100 mg) were mixed with ethanol (4 mL), spiked with vitamin K1(25) internal standard (30 µL; 9.2 pmol/µL; #R-8002, GLSynthesis Inc., Worcester, MA, USA), and extracted twice with hexane. Combined extracts were evaporated under nitrogen gas, reconstituted in 500 µL of hexane, then purified by silica solid-phase extraction (SPE; Bond Elut, 500 mg; #12113036; Agilent Technologies, Santa Clara, CA, USA). Cartridges were conditioned under vacuum with 4 mL diethyl ether/hexane (3.5:96.5, v/v) followed by 4 mL hexane. Samples were then loaded onto the cartridges, washed with 4 mL hexane, and eluted with 8 mL diethyl ether/hexane (3.5:96.5, v/v). Eluates were dried, reconstituted in dichloromethane:methanol (3:10, v/v), and filtered through a 4 mm, 0.45 µm PVDF syringe filters (#SLHVR04NL; Millex-HV Durapore^®^, Millipore Sigma, Sigma-Aldrich Inc., St. Louis, MO, USA) prior to analysis. Samples were analyzed by APCI-LC-MS in positive-ion mode using a Shimadzu LCMS-2020. Chromatographic separation was performed at 0.3 mL/min using a Phenomenex Kinetex C18 column (100 × 2.1 mm, 2.6 µm, 100 Å; 25 °C) with a step-gradient of 5.5 mM acetic acid in methanol (mobile phase A) and dichloromethane (mobile phase B), as follows: 10% solvent B at 0 min; at 6 min, solvent B was increased to 30% (flow rate = 0.35 mL/min); at 9 min, solvent B was returned to 10% for column re-equilibration; total run time was 12 min. The APCI interface temperature was set to 300 °C, with the desolvation line and heat block temperatures set to 200 °C each, and the nebulizing and drying gas flow rates set to 2.0 and 5.0 L/min, respectively. Single ion monitoring was performed at *m/z* 451.3–471.3 (d_0_–d_20_) and *m/z* 521.7 for the internal standard. VK1 concentrations were calculated from summed isotopomer peak areas relative to the internal standard and external calibration curves.

For both α-T and VK1, chromatographic data were acquired and processed using LabSolutions version 5.97 SP1 (Shimadzu Corporation, Kyoto, Japan). Peak areas were determined using the LabSolutions Auto Integration function with consistent integration settings across standards, blanks, and study samples. All chromatograms were visually inspected, and integrations were manually adjusted when automatic integration did not correctly identify the analyte peak or establish the baseline. The limits of detection (LOD) and quantification (LOQ) were calculated from low-concentration calibration curves as 3.3 and 10 times the residual standard deviation of the regression divided by the calibration-curve slope, respectively (Ermer, 2025). Recovery was calculated by comparing internal-standard peak areas (tocol for α-T and vitamin K1(25) for VK1) in study samples with the mean internal-standard peak area of the corresponding calibration standards. Conventional analyte spike-recovery assessment was not feasible because the samples contained both unlabeled and intrinsically deuterated α-T and VK1 isotopomers. Intra-assay CVs were calculated within each analytical run, and inter-assay CVs were calculated across run means.

The α-T calibration curve ranged from 3.75 to 240 pmol/µL and was fitted using unweighted linear regression of the analyte-to-internal-standard peak-area ratio against concentration (*R*^2^ = 0.9911). The LOD and LOQ were 107 and 323 fmol injected, respectively. Blank injections were included throughout the analytical runs to monitor background signal and carryover; no quantifiable α-T peaks were observed. Mean recovery was 99.2% across three runs (range: 97.8%–101.1%). Intra- and inter-assay CVs were 2.3%–2.9% and 1.7%, respectively.

The VK1 calibration curve ranged from 2.54 to 40.61 pmol/µL and was fitted using unweighted linear regression of the analyte-to-internal-standard peak-area ratio against concentration (*R*^2^ = 0.9997). The LOD and LOQ were 82 and 250 fmol injected, respectively. Blank injections were included throughout the analytical runs to monitor background signal and carryover; no quantifiable VK1 peaks were observed. Mean recovery was 100.0% across three runs (range: 98.3%–103.3%). Intra-assay CVs ranged from 8.6% to 9.9%, and the inter-assay CV was 2.9%.

### Statistical analysis

All statistical analyses were performed using GraphPad Prism (version 10; San Diego, CA, USA). Data are reported as mean ± SEM. Each hydroponic growth tray was treated as an experimental unit, and all outcomes were analyzed using one tray-level value at each harvest. For biomass, the five subsample measurements were averaged to obtain one tray-level value; for α-T and VK1, the five subsamples were combined to generate one tray-level analytical sample. This yielded *n* = 3 tray-level observations per condition at each harvest time.

The d_0_–d_2_ and d_3_–d_20_ mass ranges were classified based on the predicted natural-isotope distributions of α-T and VK1. Based on the molecular formulas of α-T (C_29_H_50_O_2_) and VK1 (C_31_H_46_O_2_) and the reported natural abundances of ^13^C (1.07%), ^2^H (0.0115%), ^17^O (0.038%), and ^18^O (0.205%) (Midani et al., 2017), the predicted relative abundances of the d_0_, d_1_, and d_2_ mass isotopomers were approximately 72.4%, 23.2%, and 3.9% for α-T and 70.9%, 24.2%, and 4.3% for VK1, respectively. Together, d_0_–d_2_ accounted for approximately 99.5% of the predicted natural-isotope distribution of α-T and 99.4% of that of VK1, whereas d_3_ accounted for approximately 0.5% of each compound. Therefore, the d_0_–d_2_ region, to which naturally occurring isotopes predominantly contribute in unlabeled compounds, was excluded, and summed d_3_–d_20_ relative abundance was used to estimate deuterium enrichment.

Two-way repeated-measures ANOVA was used to assess the effects of isotopic labeling condition and harvest time and their interaction, followed by Tukey’s *post hoc* test where appropriate. For isotopomer enrichment analyses, summed d_3_–d_20_ relative abundance was used as the enrichment endpoint for α-T and VK1 and was logit-transformed before analysis using ln[Y/(100 − Y)], where Y represents relative abundance expressed as a percentage. Control-adjusted enrichment was calculated within each experiment by subtracting the summed d_3_–d_20_ relative abundance in the non-D_2_O control from that in the corresponding D_2_O-grown tray. Inter-run precision was evaluated by calculating inter-experiment coefficients of variation (CV%) for the summed relative abundance of labeled isotopomers (d_3_–d_20_) in D_2_O-grown spinach at each harvest time.

## Results

### Spinach leaf biomass

Spinach leaf biomass over time across both labeling conditions (nominal 30% [v/v] D_2_O and non-D_2_O) is summarized in **Table 2**. No significant labeling condition × harvest time interaction was observed (*p* = 0.1474). The estimated mean difference between D_2_O and non-D_2_O conditions was 0.59 g (95% CI: −0.67 to 1.85 g; *p* = 0.2638), indicating no significant main effect of labeling condition. A significant main effect of harvest time was detected (*p* = 0.0001). Across labeling conditions, leaf biomass was greater at Week 5 than at Week 4 (mean difference: 0.67 g; 95% CI: 0.07–1.26 g; *p* = 0.0296), at Week 6 than at Week 5 (mean difference: 0.99 g; 95% CI: 0.40–1.59 g; *p* = 0.0035), and at Week 6 than at Week 4 (mean difference: 1.66 g; 95% CI: 1.07–2.25 g; *p* = 0.0001).

**Table 2:** Spinach leaf biomass (g) at Weeks 4–6 post-germination in 30% (v/v) D_2_O vs non-D_2_O conditions^1^.

Harvest time	30% (v/v) D_2_O	Non-D_2_O
Week 4^c^	2.73 ± 0.32	2.67 ± 0.21
Week 5^b^	3.81 ± 0.38	2.92 ± 0.48
Week 6^a^	4.77 ± 0.08	3.95 ± 0.51

D_2_O, deuterium oxide.

^1^ Mean ± SEM total fresh leaf mass of spinach grown in nominal 30% (v/v) D_2_O and non-D_2_O hydroponic nutrient solution at Weeks 4, 5, and 6 (n = 3 per condition per harvest). Five subsamples were collected from each tray at each harvest time. For each subsample, the combined weight of six plants was measured to account for spatial variability in growth within the tray and to better represent leaf biomass at each harvest. The five subsample weights were averaged to obtain one tray-level mean.

Statistical analysis was performed using two-way repeated-measures ANOVA with labeling condition and harvest time as factors. There was no significant interaction between labeling condition and harvest time (*p* > 0.05) and no significant main effect of labeling condition (*p* > 0.05). However, a significant main effect of harvest time was observed (*p* < 0.001). Harvest times not sharing a common superscript (a–c) differ significantly when averaged across labeling conditions (*p* < 0.05).

### d_x_-α-T content and isotopomer distribution

Representative LC–MS chromatograms for α-T isotopomers (d_0_–d_20_) from D_2_O- and non-D_2_O-grown spinach are shown in **Figure 4**. The relative isotopomer distributions of α-T in spinach grown in 30% (v/v) D_2_O and non-D_2_O hydroponic nutrient solution from Experiment 1 at each harvest timepoint are shown in **Figures 5A–C**. Corresponding distributions from Experiments 2 and 3 are shown in **Supplementary Figures S1**, **S2**.

**Figure 4:**
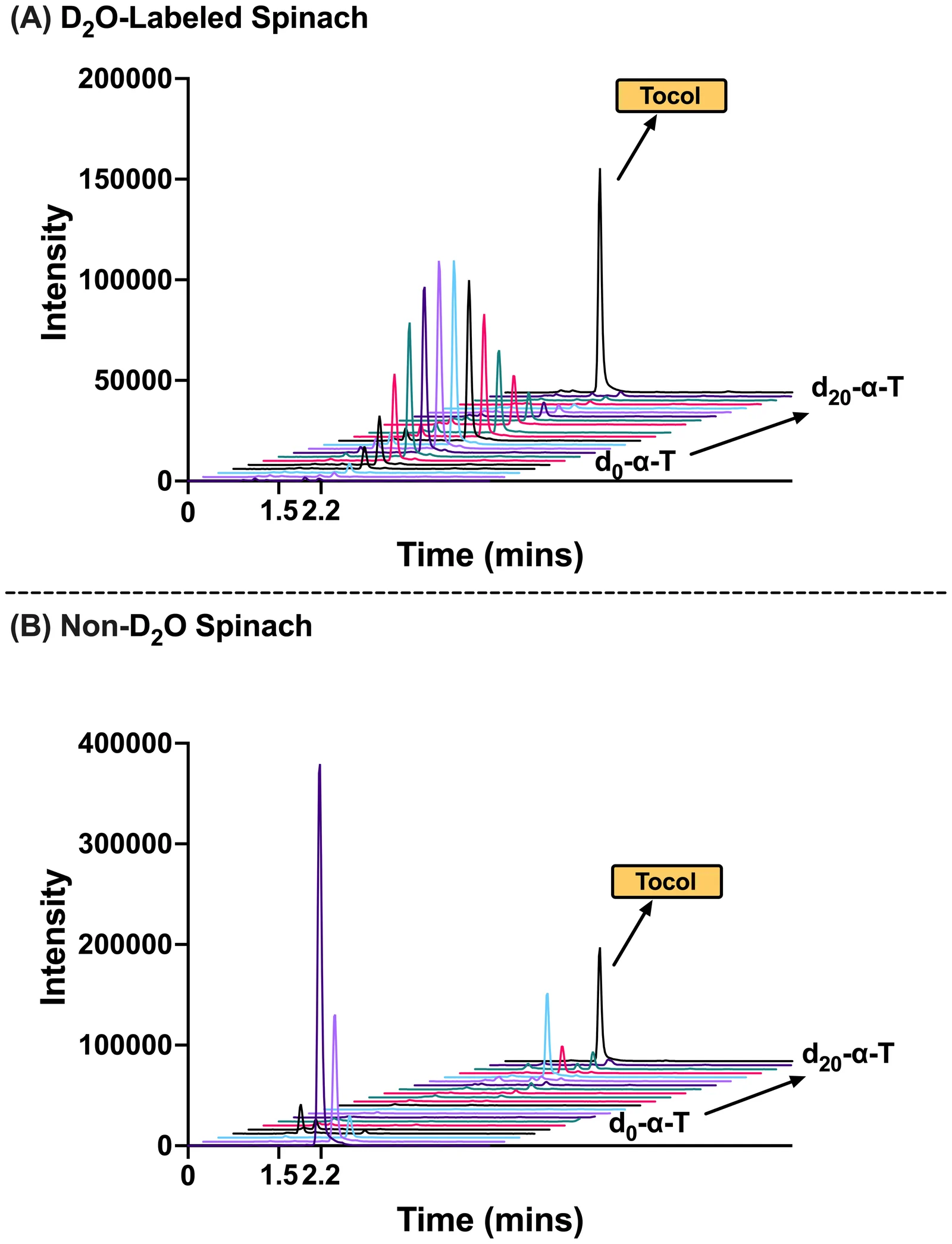
Representative LC–MS chromatograms of α-tocopherol (α-T) in spinach grown under nominal 30% (v/v) deuterium oxide (D_2_O) and non-D_2_O conditions. **(A)** Spinach grown in 30% (v/v) D_2_O and harvested at week 6 of growth. **(B)** Spinach grown under non-D_2_O conditions and harvested at week 6 of growth. Unlabeled (d_0_) and deuterium-labeled (d_1_–d_20_) α-T were quantified using selected-ion monitoring at m/z 429 for d_0_ and m/z 430–449 (1 amu increments) for d_1_–d_20_. α-T eluted at 2.2 min. The internal standard, tocol, was monitored at m/z 387.3 and eluted at 1.5 min.

**Figure 5:**
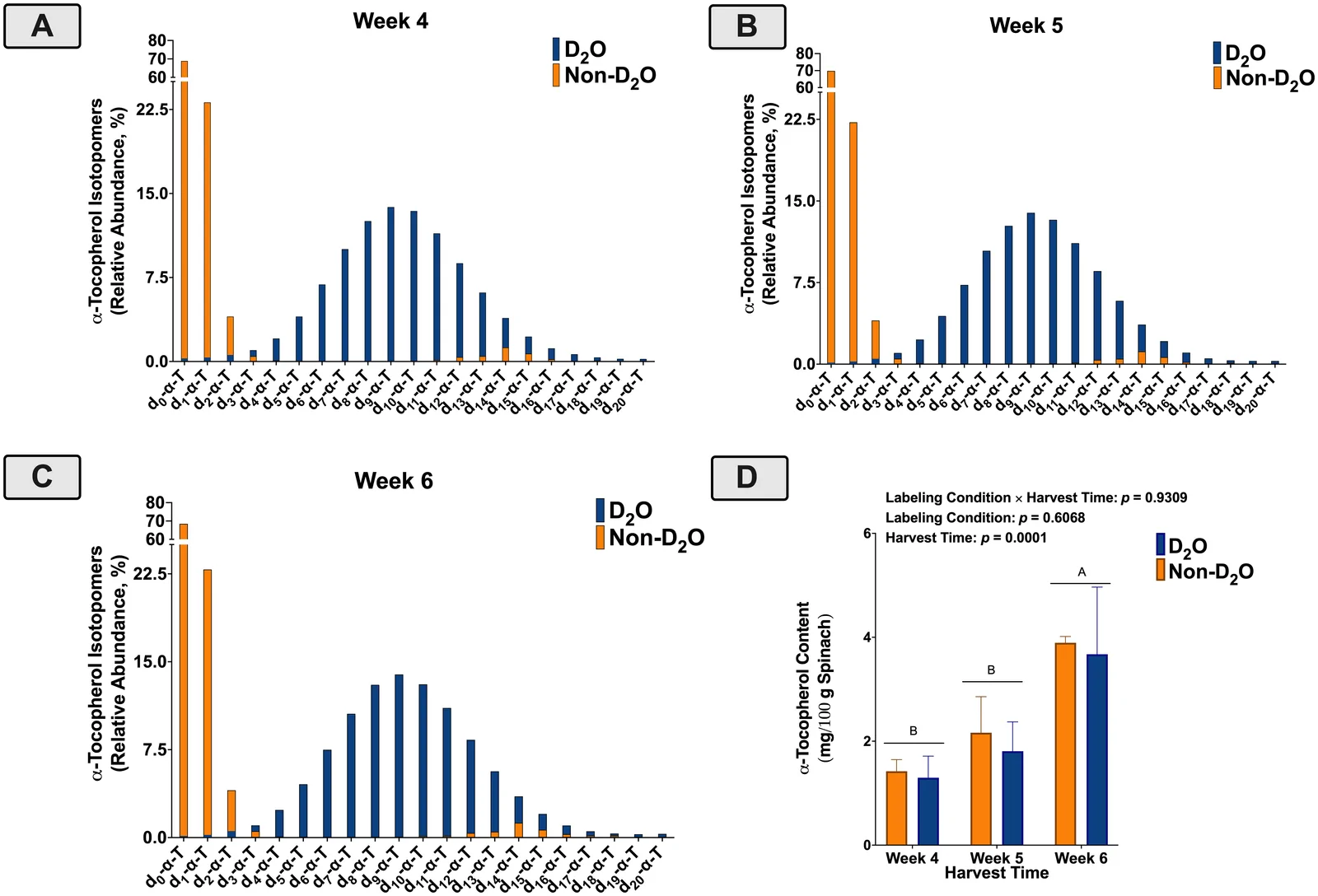
Isotopomer distribution and α-tocopherol (α-T) content in spinach grown under nominal 30% (v/v) deuterium oxide (D_2_O) and non-D_2_O conditions. **(A–C)** Isotopomer distribution of d_x_-α-T in spinach grown in 30% (v/v) D_2_O and non-D_2_O hydroponic nutrient solution, harvested at week 4, week 5, and week 6. Unlabeled (d_0_) and labeled (d_1_–d_20_) α-T were quantified using selected ion monitoring at a mass-to-charge ratio (m/z) of 429 for d_0_ and m/z 430–449 (1 amu increments) for d_1_–d_20_. Each bar represents the relative abundance of each isotopomer in the tray-level analytical samples from experiment 1, calculated as the peak area of each isotopomer relative to the total peak area of all isotopomers (d_0_-d_20_). **(D)** Mean ± SEM of α-T content in 100 g of spinach grown under D_2_O and non-D_2_O labeling conditions at Weeks 4, 5, and 6 (n = 3 trays per condition at each harvest time). Statistical analysis was performed using two-way repeated-measures ANOVA with factors of labeling condition and harvest time. Tukey *post-hoc* comparisons were conducted for the main effect of time, with significant differences between time points indicated by different superscripts.

Analysis of logit-transformed summed d_3_–d_20_ relative abundance showed no significant labeling condition × time interaction (*p* = 0.4273) or main effect of harvest time (*p* = 0.0760), but showed a significant main effect of labeling condition (*p* = 0.0002). Across harvest times, the mean difference calculated from the untransformed d_3_–d_20_ relative abundance values between D_2_O-grown and non-D_2_O-grown spinach was 94.82 percentage points (95% CI: 94.19–95.45). In D_2_O-grown spinach, d_3_–d_20_ accounted for 98.7 ± 0.1%, 99.0 ± 0.0%, and 99.0 ± 0.1% of the total α-T isotopomer pool at Weeks 4, 5, and 6, respectively. In contrast, d_3_–d_20_ isotopomers in non-D_2_O-grown spinach accounted for only 3.6 ± 0.3%, 4.0 ± 0.4%, and 4.6 ± 0.1% of the total α-T isotopomer pool at Weeks 4, 5, and 6, respectively. After subtracting the summed d_3_–d_20_ relative abundance observed in non-D_2_O-grown spinach, the control-adjusted summed d_3_–d_20_ relative abundance in D_2_O-grown spinach was 95.1 ± 0.4%, 95.0 ± 0.4%, and 94.4 ± 0.1% at Weeks 4, 5, and 6, respectively. Across all harvest times, the dominant isotopomer within labeled spinach was d_9_-α-T, accounting for 14% of total α-T abundance. Inter-experiment CVs for summed d_3_–d_20_ relative abundance in D_2_O-grown spinach were 0.3%, 0.1%, and 0.2% at Weeks 4, 5, and 6, respectively. Inter-experiment CVs for the relative abundance of each individual α-T isotopomer are reported in **Supplementary Table S2**.

Mean α-T content across harvest times and labeling conditions is shown in **Figure 5D**. There was no interaction between labeling condition and time (*p* = 0.9309). The estimated mean difference between non-D_2_O and D_2_O conditions was 0.23 mg/100 g (95% CI: −0.93 to 1.40 mg/100 g; *p* = 0.6068), indicating no significant main effect of labeling condition. However, a main effect of time was observed (*p* = 0.0001). Across labeling conditions, α-T content was greater at Week 6 than at Week 4 (mean difference: 2.42 mg/100 g; 95% CI: 1.55–3.30 mg/100 g; *p* = 0.0001) and Week 5 (mean difference: 1.80 mg/100 g; 95% CI: 0.92–2.67 mg/100 g; *p* = 0.0009), whereas Weeks 4 and 5 did not differ (mean difference: 0.63 mg/100 g; 95% CI: −0.25 to 1.50 mg/100 g; *p* = 0.1628). These results support that intrinsic D_2_O labeling did not alter α-T accumulation while yielding similar isotopomer distributions across the three experiments.

### d_x_-VK1 content and isotopomer distribution

Representative LC–MS chromatograms of VK1 isotopomers (d_0_–d_20_) from D_2_O- and non-D_2_O-grown spinach are shown in **Figure 6**, and isotopomer distributions from Experiment 1 across harvest times are in **Figures 7A–C**. Corresponding distributions from Experiments 2 and 3 are shown in **Supplementary Figures S3**, **S4**.

**Figure 6:**
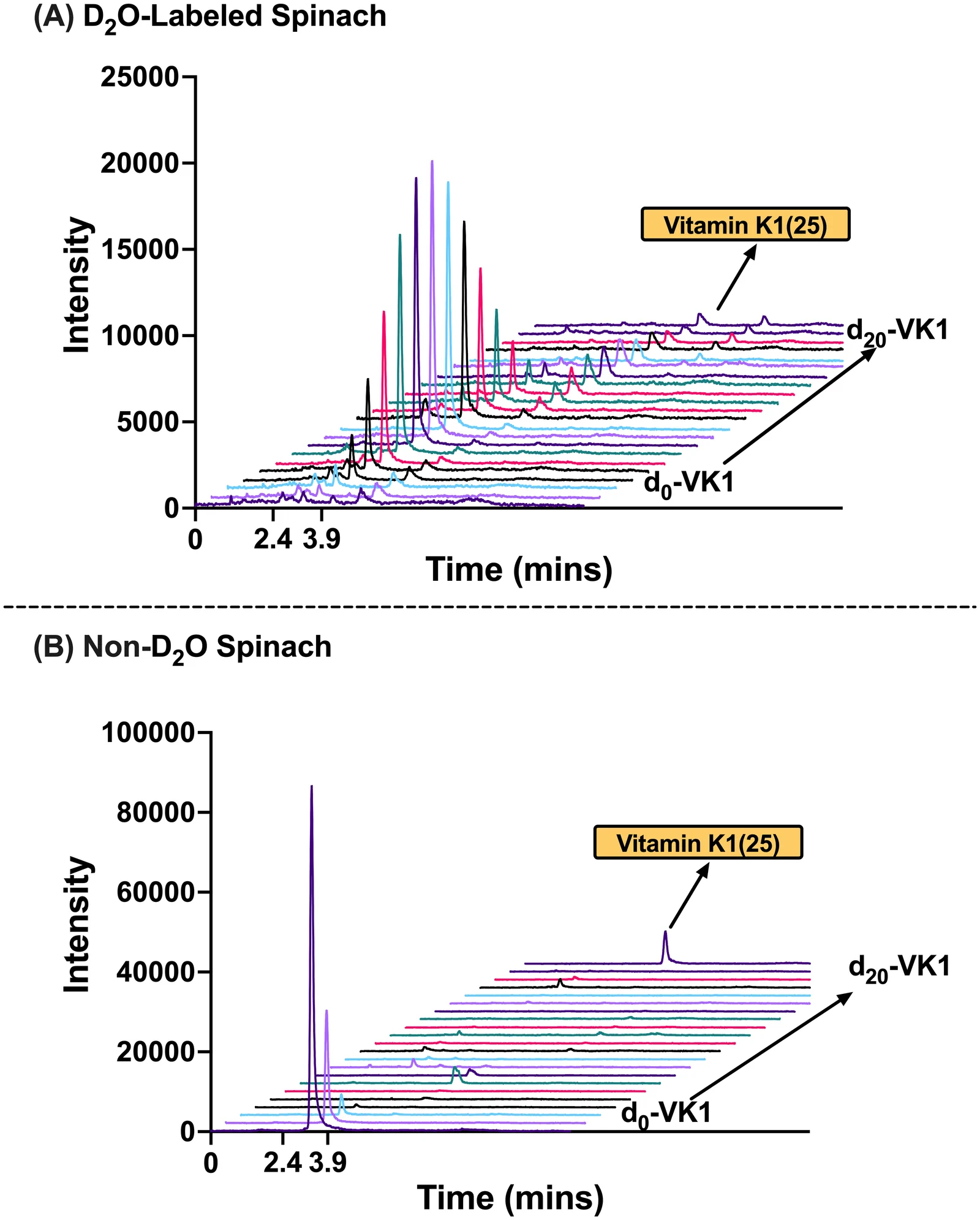
Representative LC–MS chromatograms of phylloquinone (VK1) in spinach grown under nominal 30% (v/v) deuterium oxide (D_2_O) and non-D_2_O conditions. **(A)** Spinach grown in 30% (v/v) D_2_O and harvested at week 6 of growth. **(B)** Spinach grown under non-D_2_O conditions and harvested at week 6 of growth. Unlabeled (d_0_) and deuterium-labeled (d_1_–d_20_) VK1 were quantified using selected ion monitoring at a mass-to-charge ratio (m/z) of 451.3 for d_0_ and m/z 452.3–471.3 (1 amu increments) for d_1_–d_20_ eluted at 2.4 min. The internal standard, vitamin K1(25), was monitored at m/z 521.7 and eluted at 3.9 min.

**Figure 7:**
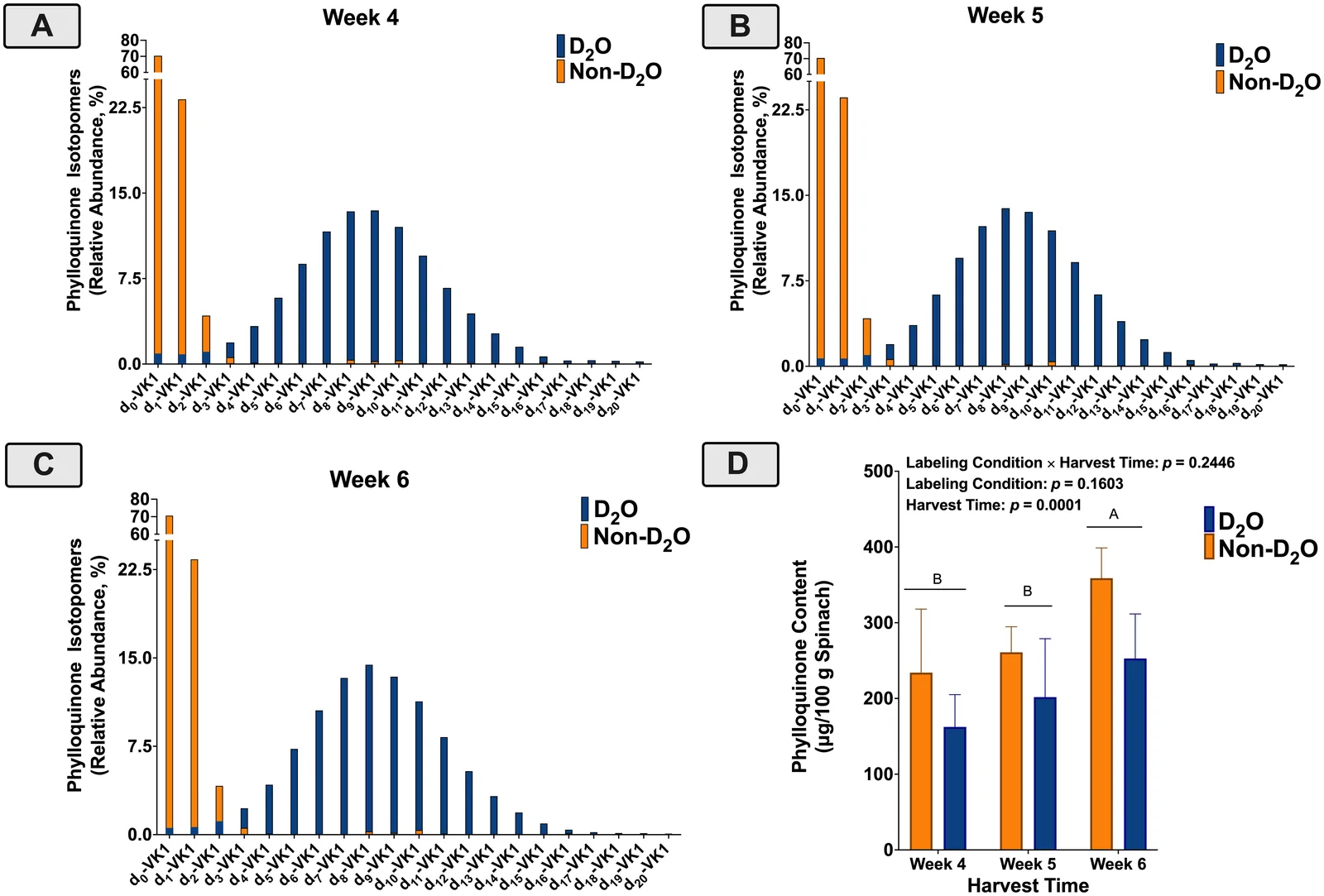
Isotopomer distribution and phylloquinone (VK1) content in spinach grown under nominal 30% (v/v) deuterium oxide (D_2_O) and non-D_2_O conditions. **(A–C)** Isotopomer distribution of d_x_-VK1 in spinach grown in nominal 30% (v/v) D_2_O and non-D_2_O hydroponic nutrient solution, harvested at Week 4, Week 5, and Week 6. Unlabeled (d_0_) and deuterium-labeled (d_1_–d_20_) VK1 were quantified using selected ion monitoring at a mass-to-charge ratio (m/z) of 451.3 for d_0_ and m/z 452.3–471.3 (1 amu increments) for d_1_–d_20_. Each bar represents the relative abundance of each isotopomer in the tray-level analytical samples from experiment 1, calculated as the peak area of each isotopomer relative to the total peak area of all isotopomers (d_0_–d_20_). **(D)** Mean ± SEM of VK1 content in 100 g of spinach grown under D_2_O and non-D_2_O labeling conditions at Weeks 4, 5, and 6 (n = 3 trays per condition at each harvest time) Statistical analysis was performed using two-way repeated-measures ANOVA with factors of labeling condition and harvest time. Tukey *post-hoc* comparisons were conducted for the main effect of time, with significant differences between time points indicated by different superscripts.

Analysis of logit-transformed summed d_3_–d_20_ relative abundance showed no labeling condition × time interaction (*p* = 0.4575) or main effect of harvest time (*p* = 0.2160), but showed a significant main effect of labeling condition (*p* = 0.0008). Across harvest times, the mean difference calculated from the untransformed summed d_3_–d_20_ relative abundance values was 95.43 percentage points, with higher relative abundance in D_2_O-grown spinach than in non-D_2_O-grown spinach (95% CI: 94.44–96.42). In D_2_O-grown spinach, d_3_–d_20_ accounted for 97.0 ± 0.2%, 97.5 ± 0.1%, and 97.5 ± 0.0% of total VK1 abundance at Weeks 4, 5, and 6, respectively. In contrast, d_3_–d_20_ isotopomers in non-D_2_O-grown spinach accounted for only 2.1 ± 0.6%, 1.8 ± 0.3%, and 1.9 ± 0.2% of the total VK1 isotopomer pool at Weeks 4, 5, and 6, respectively. After subtracting the summed d_3_–d_20_ relative abundance observed in non-D_2_O-grown spinach, the control-adjusted summed d_3_–d_20_ relative abundance in D_2_O-grown spinach was 95.0 ± 0.8%, 95.7 ± 0.2%, and 95.7 ± 0.2% at Weeks 4, 5, and 6, respectively. The dominant labeled isotopomer was d_9_ at Week 4 (13.5 ± 0.2%) and d_8_ at Week 5 (13.9 ± 0.1%) and Week 6 (14.4 ± 0.1%). Inter-experiment CVs for summed d_3_–d_20_ relative abundance in D_2_O-grown spinach were 0.4%, 0.1%, and 0.1% at Weeks 4, 5, and 6, respectively. Inter-experiment CVs for the relative abundance of each individual VK1 isotopomer are reported in **Supplementary Table S3**.

Mean VK1 content across harvest times and labeling conditions showed no significant labeling condition × time interaction (*p* = 0.2446; **Figure 7D**). In addition, there was no significant main effect of labeling condition (mean difference between non-D_2_O and D_2_O conditions: 78.97 μg/100 g; 95% CI: −48.39 to 206.3 μg/100 g; *p* = 0.1603). However, a significant main effect of harvest time was observed (*p* = 0.0001). Across labeling conditions, VK1 content was greater at Week 6 than at Week 4 (mean difference: 107.5 μg/100 g; 95% CI: 69.84–145.2 μg/100 g; *p* < 0.0001) and Week 5 (mean difference: 74.45 μg/100 g; 95% CI: 36.75–112.2 μg/100 g; *p* = 0.0012), whereas Weeks 4 and 5 did not differ (mean difference: 33.09 μg/100 g; 95% CI: −4.61 to 70.79 μg/100 g; *p* = 0.0836). These findings indicate that, similar to α-T, VK1 accumulation was not significantly affected by D_2_O labeling, while isotopomer enrichment remained stable across the three experiments.

## Discussion

Our results indicate that the hydroponic growth conditions developed in this study are sufficient to allow the production of spinach producing deuterated vitamin E and phylloquinone at both concentrations and isotopomeric distributions with potential application in future plant fluxomic or dietary nutrition/incorporation studies. The static D_2_O-labeling protocol described here provides a method for generating intrinsically labeled spinach suitable for bioavailability and metabolic tracing applications without the specialized infrastructure required for recirculating hydroponic or sealed atmospheric systems. The labeled spinach produced using this protocol was evaluated in a human α-T bioavailability study (Rezaei et al., 2026). Across three independent experiments conducted in the same laboratory, the protocol produced similar isotopomer distributions for both α-T and VK1, dominated by d_8_–d_9_ isotopomer species, which could inform downstream bioanalytical quantification in future studies. Within the evaluated harvest window, extending growth to Week 6 was associated with greater leaf biomass and higher α-T and VK1 content per 100 g of spinach, which may improve the practical efficiency of producing labeled plant material. Importantly, neither α-T nor VK1 content was significantly affected by D_2_O labeling under the evaluated conditions. Collectively, these findings demonstrate that the hydroponic D_2_O labeling method presented here provides a detailed framework for producing intrinsically labeled spinach under the evaluated conditions and establishes a methodological foundation for future metabolic tracing studies of nutrient bioavailability from intact plant food matrices.

The isotopomer distributions observed in this study confirm successful intrinsic labeling of spinach-derived α-T and VK1 using 30% (v/v) D_2_O. As expected, spinach grown without D_2_O was dominated by naturally abundant isotopomers, whereas D_2_O-grown spinach exhibited a pronounced shift toward labeled isotopomers across all harvest times. The dominant labeled isotopomer was d_9_ for α-T and d_8_–d_9_ for VK1, and these distributions were stable across independent experiments. These labeling patterns are consistent with prior intrinsic D_2_O-labeling studies in green leafy vegetables conducted at comparable enrichment levels (**Table 1**) (Traber et al., 2015; Ellis et al., 2019; Erkkilä et al., 2004). For example, collard greens grown with 31 atom% D_2_O exhibited dominant isotopomers of d_11_ for α-T and d_9_–d_10_ for VK1 (**Table 1**) (Ellis et al., 2019; Erkkilä et al., 2004). Taken together, existing studies and the present findings indicate that intrinsic labeling with ~30% D_2_O reliably produces dominant isotopomers in the ranges of d_9_–d_11_ for α-T and d_8_–d_10_ for VK1 in leafy greens. These predictable isotopomer profiles are critical for downstream bioanalytical applications. Indeed, identification of dominant isotopomers in plant tissue enables targeted metabolomics for human plasma analysis, thereby improving analytical sensitivity, reducing noise from low-abundance species, and facilitating more quantification of nutrient bioavailability in human studies.

Harvest timing has direct implications for bioavailability studies, as it determines the amount of plant material required to deliver target nutrient doses. Maturation of spinach to Week 6 resulted in the highest leaf biomass and the highest α-T and VK1 content per 100 g of spinach. This finding has direct implications for bioavailability studies, as harvest timing determines the amount of plant material required to deliver target nutrient doses. Although no prior study has examined α-T and VK1 content in intrinsically labeled spinach, a previous D_2_O-labeling study using a different spinach cultivar (Melody) harvested plants at Week 4 for lutein bioavailability assessment (Lienau et al., 2003). In the present study, actual tray yields were not directly measured because the same trays were sampled at Weeks 4, 5, and 6 rather than separate trays being harvested in full at each time point. However, hypothetical leaf biomass for a 100-plant tray in the nominal 30% (v/v) D_2_O condition was estimated to increase from approximately 46 g at Week 4 to approximately 80 g at Week 6 by extrapolating the mean fresh mass of the six-plant subsamples to 100 plants. Importantly, most resource inputs associated with intrinsic labeling occurred during initial setup, including seed germination, tray preparation, and the initial D_2_O nutrient solution volume (~700 mL per tray). In contrast, maintenance during growth required only modest weekly replenishment (~80 mL per tray). Extending growth from Week 4 to Week 6 was associated with greater estimated labeled biomass and nutrient content without requiring additional experimental setup or a proportional increase in D_2_O use. Although this study was not designed to assess cost-effectiveness, these findings suggest that later harvests may improve the practical efficiency of producing intrinsically labeled spinach for human bioavailability studies.

Spinach tolerated the nominal 30% (v/v) D_2_O condition without impairment of leaf biomass, consistent with prior reports indicating that higher plants generally tolerate D_2_O concentrations up to ~30%, whereas higher enrichments (≥50%) are associated with growth inhibition (Katz and Crespi, 1966; Grusak, 1997). Moreover, neither α-T nor VK1 content differed significantly between labeling conditions. The combination of high isotopomer enrichment with no statistically detectable differences in biomass or nutrient content supports the practical feasibility of using nominal 30% (v/v) D_2_O to produce intrinsically labeled spinach under the evaluated conditions.

This study had several strengths. Most importantly, it addressed methodological limitations of prior intrinsic labeling studies in green leafy vegetables, which often lacked detailed descriptions of hydroponic systems or relied on protocols developed for other plant species or recirculating designs. The present work provides a comprehensive static hydroponic D_2_O-labeling protocol, including seed sterilization, growth conditions, hydroponic setup, and bioanalytical characterization, thereby facilitating adoption and adaptation across research settings. We also observed similar isotopomer distributions across three independent experiments conducted in the same laboratory under the same controlled conditions. Inclusion of non-D_2_O controls further confirmed that observed isotopomer enrichment reflected true deuterium incorporation above natural abundance. Although this study focused on α-T and VK1, intrinsic D_2_O labeling enables incorporation of deuterium into a broad range of plant-derived metabolites, suggesting that the described approach may support wider applications in metabolic tracing in plants following deuterium labeling, and in humans following labeled spinach consumption. In contrast, we acknowledge that the number of independent experimental replicates (n = 3) limits robust statistical evaluation. Thus, comparisons between D_2_O- and non-D_2_O-grown plants, despite the low inter-experiment variability observed for labeled isotopomer enrichment, should be interpreted cautiously. The effects attributed to harvest time could also have been influenced by progressive plant removal and potential changes in plant competition and canopy conditions. Future studies using separate trays assigned to each harvest time could address this limitation and isolate the effects of harvest timing. In addition, D_2_O enrichment of the nutrient solution was not measured during cultivation; therefore, changes in solution enrichment (i.e., alterations to deuterium content) over the 6-week growth period could not be evaluated. D_2_O enrichment of leaf tissue water was also not measured. Therefore, the observed α-T and VK1 isotopomer distributions should be interpreted in relation to the nominal 30% (v/v) D_2_O condition. Future studies measuring D_2_O enrichment in the nutrient solution and leaf tissue water throughout cultivation would determine whether the nominal solution enrichment is maintained, characterize the precursor enrichment within the plants, and support evaluation of deuterium flux into α-T and VK1. The protocol was developed using a single spinach cultivar under a specific set of environmental and hydroponic conditions. While our detailing of the approach provides a framework, the biomass findings and isotopomer distributions may not apply to other plant species, cultivars, or D_2_O enrichment conditions. Evaluation and optimization across additional cultivars and D_2_O concentrations are needed to determine the broader applicability of the method. Finally, scalability and cost-effectiveness for large-scale production were not evaluated and remain important considerations for future applications.

Collectively, this protocol report presents a detailed static hydroponic D_2_O-labeling protocol for generating intrinsically labeled spinach intended for future studies of α-tocopherol and phylloquinone bioavailability from intact food matrices. By addressing key methodological gaps in studies utilizing intrinsic labeling and demonstrating inter-run precision, characterizing isotopomer profiles, and establishing practical feasibility under the evaluated conditions, this work may facilitate the broader use of D_2_O-based labeling approaches for investigating plant-derived nutrients and metabolites in both plant and human nutrition research.

## Data Availability

The original contributions presented in the study are included in the article and **Supplementary Material**. Further inquiries can be directed to the corresponding authors.
