# Supplementary material for: Intrinsic deuterium labeling of spinach: a hydroponic method to enrich isotopic labeling of vitamins E and K suitable for human bioavailability studies
Source: Front Plant Sci. 2026 Oct 14;17:1915355. doi: 10.3389/fpls.2026.1915355 (PMC13713610; doi:10.3389/fpls.2026.1915355)
Supplement: Supplementary file 1 [file DataSheet1.docx]

Supplementary Materials

**Intrinsic Deuterium Labeling of Spinach: A Hydroponic Method To Enrich Isotopic Labeling Of Vitamins E and K Suitable For Human Bioavailability Studies**

**Shahabeddin Rezaei^1^, Sisi Cao^1^, Min Zeng^1^, Maria L. Antonius^1^, Allyson Q. McHenry^1^, Richard S. Bruno^1*^, Joshua J. Blakeslee^2*^**

^1^Human Nutrition Program, Department of Human Sciences, The Ohio State University

^2^Department of Horticulture and Crop Science, The Ohio State University

*** Co-Corresponding Authors:**
Joshua J. Blakeslee: [blakeslee.19@osu.edu](mailto:blakeslee.19@osu.edu)

Richard S. Bruno: [bruno.27@osu.edu](mailto:bruno.27@osu.edu)

| **Supplementary Table S1.** Individual experiment-level tray means for spinach leaf biomass at Weeks 4–6 under nominal 30% (v/v) D₂O and non-D₂O conditions.^1^ | | | | | | |
| --- | --- | --- | --- | --- | --- | --- |
| **Harvest Time** | **Non-D_2_O** | | | **30% (v/v) D_2_O** | | |
|  | **Experiment 1** | **Experiment 2** | **Experiment 3** | **Experiment 1** | **Experiment 2** | **Experiment 3** |
| Week 4 | 2.26 | 2.94 | 2.80 | 2.86 | 3.20 | 2.12 |
| Week 5 | 2.00 | 3.62 | 3.14 | 4.22 | 4.16 | 3.04 |
| Week 6 | 3.04 | 4.00 | 4.80 | 4.84 | 4.86 | 4.60 |
| **Abbreviations:** D_2_O, deuterium oxide  ^1^Each value represents one tray-level mean calculated by averaging the five subsample weights collected from the corresponding tray at each harvest time. Each subsample represented the combined fresh leaf mass of six plants. Each experiment included one nominal 30% (v/v) D_2_O tray and one non-D_2_O tray. | | | | | | |

| **Supplementary Table S2.** Inter-experiment CVs (%) for the relative abundances of individual α‑T mass isotopomers in D_2_O-grown spinach at Weeks 4–6.^1^ | | | |
| --- | --- | --- | --- |
| **Isotopomer** | **Week 4** | **Week 5** | **Week 6** |
| d_0_-α-T | 33.3 | 15.7 | 9.4 |
| d_1_-α-T | 25.1 | 6.1 | 13.1 |
| d_2_-α-T | 11.6 | 7.0 | 20.1 |
| d_3_-α-T | 9.4 | 12.3 | 24.4 |
| d_4_-α-T | 11.1 | 12.0 | 21.9 |
| d_5_-α-T | 10.5 | 9.6 | 18.5 |
| d_6_-α-T | 8.3 | 8.3 | 15.0 |
| d_7_-α-T | 5.8 | 5.7 | 9.8 |
| d_8_-α-T | 3.7 | 4.0 | 5.0 |
| d_9_-α-T | 1.1 | 0.8 | 1.1 |
| d_10_-α-T | 1.6 | 2.5 | 5.1 |
| d_11_-α-T | 3.7 | 4.4 | 10.1 |
| d_12_-α-T | 6.0 | 7.8 | 15.3 |
| d_13_-α-T | 9.4 | 10.0 | 20.7 |
| d_14_-α-T | 10.7 | 12.0 | 24.8 |
| d_15_-α-T | 13.5 | 14.8 | 30.4 |
| d_16_-α-T | 13.6 | 14.0 | 28.3 |
| d_17_-α-T | 16.1 | 11.9 | 22.5 |
| d_18_-α-T | 17.8 | 10.5 | 12.3 |
| d_19_-α-T | 9.6 | 4.4 | 5.5 |
| d_20_-α-T | 8.4 | 9.8 | 8.6 |
| **Abbreviations:** α-T, α-tocopherol; CV, coefficient of variation; D_2_O, deuterium oxide  ^1^ At each harvest time, each experiment contributed one D_2_O-grown tray-level analytical sample. CVs were calculated across the three experiments (n = 3) as (standard deviation/mean) × 100. | | | |

| **Supplementary Table S3.** Inter-experiment CVs (%) for the relative abundances of individual VK1 mass isotopomers in D_2_O-grown spinach at Weeks 4–6.^1^ | | | |
| --- | --- | --- | --- |
| **Isotopomer** | **Week 4** | **Week 5** | **Week 6** |
| d_0_-VK1 | 16.3 | 5.4 | 7.7 |
| d_1_-VK1 | 11.4 | 5.4 | 15.9 |
| d_2_-VK1 | 15.8 | 1.0 | 5.1 |
| d_3_-VK1 | 14.3 | 11.3 | 9.8 |
| d_4_-VK1 | 9.3 | 9.1 | 9.9 |
| d_5_-VK1 | 5.5 | 8.4 | 8.2 |
| d_6_-VK1 | 3.3 | 7.3 | 6.4 |
| d_7_-VK1 | 1.3 | 5.6 | 5.6 |
| d_8_-VK1 | 1.1 | 1.2 | 1.4 |
| d_9_-VK1 | 2.7 | 1.1 | 2.3 |
| d_10_-VK1 | 4.4 | 3.7 | 4.0 |
| d_11_-VK1 | 5.4 | 7.2 | 7.2 |
| d_12_-VK1 | 7.8 | 11.1 | 13.2 |
| d_13_-VK1 | 5.4 | 10.6 | 11.9 |
| d_14_-VK1 | 2.6 | 7.2 | 7.4 |
| d_15_-VK1 | 7.9 | 0.8 | 3.2 |
| d_16_-VK1 | 16.4 | 30.9 | 38.1 |
| d_17_-VK1 | 22.6 | 33.3 | 33.3 |
| d_18_-VK1 | 51.5 | 31.0 | 14.3 |
| d_19_-VK1 | 64.3 | 47.4 | 23.1 |
| d_20_-VK1 | 82.6 | 44.4 | 11.1 |
| **Abbreviations:** VK1, phylloquinone; CV, coefficient of variation; D_2_O, deuterium oxide  ^1^ At each harvest time, each experiment contributed one D_2_O-grown tray-level analytical sample. CVs were calculated across the three experiments (n = 3) as (standard deviation/mean) × 100. | | | |

**Supplementary Figure S1.** Isotopomer distributions of α-tocopherol (α-T) from Experiment 2. Spinach was grown under nominal 30% (v/v) deuterium oxide (D_2_O) and non-D_2_O conditions. (A–C) α-T isotopomer distributions at Weeks 4, 5, and 6, respectively. Each bar represents one tray-level relative-abundance value for the indicated labeling condition, calculated as the peak area of each isotopomer relative to the total peak area of all isotopomers (d_0_–d_20_).

**Supplementary Figure S2.** Isotopomer distributions of α-tocopherol (α-T) from Experiment 3. Spinach was grown under nominal 30% (v/v) deuterium oxide (D_2_O) and non-D_2_O conditions. (A–C) α-T isotopomer distributions at Weeks 4, 5, and 6, respectively. Each bar represents one tray-level relative-abundance value for the indicated labeling condition, calculated as the peak area of each isotopomer relative to the total peak area of all isotopomers (d_0_–d_20_).

**Supplementary Figure S3. Isotopomer distributions of phylloquinone (VK1) from Experiment 2. Spinach was grown under nominal 30% (v/v) deuterium oxide (**D_2_O**) and non-**D_2_O **conditions. (A–C) VK1 isotopomer distributions at Weeks 4, 5, and 6, respectively. Each bar represents one tray-level relative-abundance value for the indicated labeling condition, calculated as the peak area of each isotopomer relative to the total peak area of all isotopomers (**d_0_–d_20_**).**

**Supplementary Figure S4. Isotopomer distributions of phylloquinone (VK1) from Experiment 3. Spinach was grown under nominal 30% (v/v) deuterium oxide (**D_2_O**) and non-**D_2_O **conditions. (A–C) VK1 isotopomer distributions at Weeks 4, 5, and 6, respectively. Each bar represents one tray-level relative-abundance value for the indicated labeling condition, calculated as the peak area of each isotopomer relative to the total peak area of all isotopomers (**d_0_–d_20_**).**
